# Supplementary material for: Longitudinal 1H NMR-Based Metabolomics in Saliva Unveils Signatures of Transition from Acute to Post-Acute Phase of SARS-CoV-2 Infection
Source: Viruses. 2024 Nov 13;16(11):1769. doi: 10.3390/v16111769 (PMC11598993; doi:10.3390/v16111769)
Supplement: Supplementary file 1 [file viruses-16-01769-s001.zip › viruses-3232964-supplementary.pdf]

Supplementary material

Table S1. Assigned metabolites in saliva samples in the non-COVID and COVID-19 groups.

Metabolites	Chemical Shift, ppm	T1 non COVID Median p25 - p75%	T2 non-COVID Median p25 - p75%	p-value[1]	T1 COVID-19 Median p25 -p 75%	T2 COVID-19 Median p25 -p 75%	p-value[1]
**2-hydroxybutyrate**	**0.89**	1.66 x 10⁻³<br>1.09 x 10⁻³ - 2.45 x 10⁻³	1.52 x 10⁻³<br>1.01 x 10⁻³ - 2.21 x 10⁻³	0.9487	1.53 x 10⁻³<br>1.15 x 10⁻³ x 2.20 x10⁻³	2.00 x 10⁻³<br>1.48 x 10⁻³ - 2.86 x 10⁻³	0.0295
**Acetate**	1.90	2.50 x 10⁻³<br>2.21 x 10⁻² – 3.01 x 10⁻³	2.34 x 10⁻³<br>2.04 x 10⁻³ x 2.65 x 10⁻³	0.1026	2.68 x 10⁻³<br>2.10 x 10⁻³ x 3.03 x10⁻³	2.28 x 10⁻³<br>1.9 x 10⁻³ x 2.72 x 10⁻³	0.0289
**Acetoacetate**	2.22	1.03 x 10⁻³<br>7.75 x 10⁻⁴ - 1.22 x 10⁻³	1.08 x 10⁻³<br>8.60 x 10⁻⁴ - 1.8 x 10⁻³	0.1104	1.07 x 10⁻³<br>9.02 x 10⁻⁴ - 1.31 x 10⁻³	1.04 x 10⁻³<br>9.31 x 10⁻⁴ - 1.29 x 10⁻³	0.9594
**Alanine**	1.47	3.15 x 10⁻⁴<br>2.82 x 10⁻⁴ - 3.63 x 10⁻⁴	3.21 x 10⁻⁴<br>2.88 x 10⁻⁴-3.63 x 10⁻⁴	0.8561	3.13 x 10⁻⁴<br>2.85 x 10⁻⁴ - 3.52 x 10⁻⁴	3.26 x 10⁻⁴<br>2.48 x 10⁻⁴ x 3.52 x 10⁻⁴	0.7810
**BCAA (Isoleucine)**	**0.92***, 0.93	8.86 x 10⁻⁴<br>6.08 x 10⁻⁴ - 1.33 x 10⁻³	8.1 x 10⁻⁴<br>5.62 x 10⁻⁴ - 1.21 x 10⁻³	0.8378	8.79 x 10⁻⁴<br>6.22x 10⁻⁴-1.76 x10⁻³	1.03 x 10⁻³<br>8.27 x 10⁻⁴ - 1.55 x 10⁻³	0.0067
**Creatine and Creatinine**	**3.03**	2.62 x 10⁻⁴<br>2.06 x 10⁻⁴ - 3.35 x 10⁻⁴	2.54 x 10⁻⁴<br>2.10 x 10⁻⁴ - 3.71 x 10⁻⁴	0.8104	2.99 x 10⁻⁴<br>2.13 x 10⁻⁴ - 3.97 x 10⁻⁴	2.45 x 10⁻⁴<br>1.77 x 10⁻⁴ x 3.27 x 10⁻⁴	0.0246
**(CH3)3 Choline metabolites**	**3.19**	3.60 x 10⁻⁴<br>2.26 x 10⁻⁴ - 5.20 x 10⁻⁴	4.03 x 10⁻⁴<br>3.76x 10⁻⁴ - 5.52 x 10⁻⁴	0.1744	3.54 x 10⁻⁴<br>2.23 x 10⁻⁴ - 5.05 x 10⁻⁴	5.56 x 10⁻⁴<br>4.55 x 10⁻⁴ x 6.91 x 10⁻⁴	<0.0001
**Ethanolamine**	**3.10***, 3.12, 3.13, 3.14	1.02 x 10⁻⁴<br>7.64 x 10⁻⁵ - 1.34 x 10⁻⁴	1.19 x 10⁻⁴<br>7.35 x 10⁻⁵ - 1.51 x 10⁻⁴	0.6188	1.14 x 10⁻⁴<br>7.93 x 10⁻⁵ - 1.38 x 10⁻⁴	1.13 x 10⁻⁴<br>6.29 x 10⁻⁵ - 1.33 x 10⁻⁴	0.6752
**Fumarate**	6.50	1.0 x 10⁻⁵<br>3.90 x 10⁻⁶ - 2.1 x 10⁻⁵	1.34 x 10⁻⁵<br>5.47 x 10⁻⁵ – 2.29 ×10⁻⁵	0.4498	8.74 x 10⁻⁶<br>1.06 x 10⁻⁶-2.05 x 10⁻⁵	1.96 x 10⁻⁵<br>1.30 x 10⁻⁵ - 3.26 x 10⁻⁵	0.0044
**Sugar Regions (Mainly Glucose)**	3.53, 3.54, **3.78***, 3.81, 3.87, 3.92	1.27 x 10⁻³<br>1.16 x 10⁻³ - 1.58 x 10⁻³	1.26 x 10⁻³<br>1.03 x 10⁻³ - 1.49 x 10⁻³	0.4858	1.27 x 10⁻³<br>1.10 x 10⁻³ - 1.54 x 10⁻³	1.35 x 10⁻³<br>1.21 x 10⁻³ x 1.90 x 10⁻³	0.0221
**Histidine**	7.81	4.93 x 10⁻⁵<br>2.92 x 10⁻⁵ - 6.86 x 10⁻⁵	5.48 x 10⁻⁵<br>2.12 x 10⁻⁵ - 6.81 x 10⁻⁵	0.1083	4.9 x 10⁻⁵<br>3.50 x 10⁻⁵ – 7.1 x 10⁻⁵	2.99 x 10⁻⁵<br>1.41 x 10⁻⁵ - 6.05 x 10⁻⁵	0.0088
**Lactate**	1.31	4.38 x 10⁻⁴<br>3.56 x 10⁻⁴ - 5.98 x 10⁻⁴	3.95 x 10⁻⁴<br>3.28 x 10⁻⁴ - 4.53 x 10⁻⁴	0.0319	4.84 x 10⁻⁴<br>3.74 x 10⁻⁴ - 5.96 x 10⁻⁴	4.54 x 10⁻⁴<br>3.76 x 10⁻⁴ - 5.82 x 10⁻⁴	0.4541
**Lysine and Putrescine**	**3.04***, 3.05	1.64 x 10⁻⁴<br>1.06 x 10⁻⁴ - 1.97 x 10⁻⁴	1.68 x 10⁻⁴<br>1.18 x 10⁻⁴ - 2.23 x 10⁻⁴	0.4569	1.68 x 10⁻⁴<br>1.38 x 10⁻⁴ - 2.20 x 10⁻⁴	1.45 x 10⁻⁴<br>1.07 x 10⁻⁴ x 1.79 x 10⁻⁴	0.0282
**N-acetyl of glycoprotein**	2.05	5.38 x 10⁻⁴<br>4.31 x 10⁻⁴ - 6.03 x 10⁻⁴	5.38 x 10⁻⁴<br>4.69 x 10⁻⁴ - 6.43 x 10⁻⁴	0.5006	5.26 x 10⁻⁴<br>4.48 x 10⁻⁴- 6.44 x 10⁻⁴	5.14 x 10⁻⁴<br>4.24 x 10⁻⁴ - 5.82 x 10⁻⁴	0.3059
**Phenylalanine**	**7.27***, 7.30, 7.31, 7.38, 7.40	1.43 x 10⁻⁴<br>7.94 x 10⁻⁵- 1.91 x 10⁻⁴	1.26 x 10⁻⁴<br>8.09 x 10⁻⁵ - 1.88 x 10⁻⁴	0.4712	1.26 x 10⁻⁴<br>8.44 x 10⁻⁵ - 1.97 x 10⁻⁴	1.02 x 10⁻⁴<br>5.66 x 10⁻⁵ 1.51×10⁻⁴	0.0314
**Pyruvate**	2.31	6.96 x 10⁻⁵<br>2.63 x 10⁻⁵ - 9.85 x 10⁻⁵	7.54 x 10⁻⁵<br>9.83 x 10⁻⁵- 9.74 x 10⁻⁵	0.6420	8.68 x 10⁻⁵<br>3.52 x 10⁻⁵ x 1.12 x10⁻⁴	8.74 x 10⁻⁵<br>3.44 x 10⁻⁵ - 1.21 x 10⁻⁴	0.2533
**Succinate**	2.39	9.39 x 10⁻⁵<br>4.45 x 10⁻⁵ - 1.34 x 10⁻⁴	9.85 x 10⁻⁵<br>3.07 x 10⁻⁵ - 1.21 x 10⁻⁴	0.5860	1.01 x 10⁻⁴<br>5.39 x 10⁻⁵ - 1.30 x 10⁻⁴	1.10 x 10⁻⁴<br>6.14 x 10⁻⁵ - 1.51 x 10⁻⁴	0.4493
**Taurine**	3.23-3.25	3.25 x 10⁻⁴<br>2.60 x 10⁻⁴ - 4.33 x 10⁻⁴	3.29 x 10⁻⁴<br>2.29 x 10⁻⁴ - 4.76 x 10⁻⁴	0.7211	3.40 x 10⁻⁴<br>2.28 x 10⁻⁴ - 4.41 x 10⁻⁴	4.34 x 10⁻⁴<br>3.21 x 10⁻⁴ - 6.21 x10⁻⁴	0.0090
**Tyrosine**	**6.87***, 7.17	1.22 x 10⁻⁴<br>1.0. x10⁻⁴ - 1.70 x 10⁻⁴	1.29 x 10⁻⁴<br>1.03 x 10⁻⁴ - 2.02 x 10⁻⁴	0.5543	1.12 x 10⁻⁴<br>8.62 x 10⁻⁵ - 1.77 x 10⁻⁴	1.05 x 10⁻⁴<br>7.34 x 10⁻⁵ - 1.31 x 10⁻⁴	0.0757

*Non-overlapping peaks selected for univariate statistical analyses.
[1]Mann-Whitney test was used for comparing median values of T1 and T2 non-COVID and T1 and T2 COVID.
All metabolites were considered significant when P<0.05.

Table S2. Ranking of principal component analysis (PCA) loadings between the acute and post-acute phases of non-COVID and COVID-19 groups.

		Principal Component Analysis Loading Factors			
		T2 *vs* T1 non-COVID		T2 *vs* T1 COVID	
**Metabolites**	**Chemical Shift, ppm**	**PC 1***	**PC 2****	**PC 1***	**PC 2****
**2-hydroxybutyrate**	**0.89**	0.079274	-0.06903	0.068986	-0.15093
**Acetate**	1.90	-0.026	-0.05195	-0.038171	-0.05606
**Acetoacetate**	2.22	-0.039711	-0.046403	-0.025897	-0.025133
**Alanine**	1.47	-0.012706	0.010814	0.013185	-0.0032984
**BCAA (Isoleucine)**	**0.92**	0.060207	-0.034898	0.032132	-0.085321
**Creatine and Creatinine**	**3.03**	-0.033458	-0.033855	-0.026856	-0.039259
**$(CH_3)_3$ Choline metabolites**	**3.19**	0.023566	0.002716	0.018912	0.013349
**Ethanolamine**	**3.10**	-0.020019	-0.012804	-0.0020237	-0.0051766
**Fumarate**	6.50	0.0087362	0.0037036	0.01164	0.0041253
**Sugar Regions (Mainly Glucose)**	**3.78**	0.21245	0.00677	0.22155	0.010712
**Histidine**	7.81	-0.014119	-0.004234	-0.014226	0.0051644
**Lactate**	1.31	-0.01958	-0.054145	0.01803	-0.036505
**Lysine and Putrescine**	**3.04**	-0.030169	-0.016422	-0.025245	-0.022634
**N-acetyl of glycoprotein**	2.05	-0.039432	-0.034278	-0.022647	9.9642E-4
**Phenylalanine**	**7.27**	-0.017447	-0.013589	-0.0033437	-0.0096602
**Pyruvate**	2.31	-0.013203	0.009565	-0.0089833	0.0019721
**Succinate**	2.39	-0.016522	0.015936	-5.5702E-4	0.013677
**Taurine**	**3.23**	-0.009989	-0.020112	0.011377	-0.020906
**Tyrosine**	6.87	0.0063662	-0.0048479	0.0086296	0.0017618

*PC 1 – Principal component 1; **PC 2 – Principal component 2

Table S3. Significant changes in pathway enrichment analysis, between the acute and post-acute phases of COVID-19

Metabolic Pathways	Total[1]	Hits[2]	Expected[3]	P value[4]	Holm p[5]	FDR[6]
Glycine, serine and threonine metabolism	33	2	1.0638	3.6471E-4	0.012035	0.012035
Glutathione metabolism	28	1	1.0638	0.017074	0.54638	0.14844
Phenylalanine metabolism	8	2	1.0638	0.021006	0.65117	0.14844
Phenylalanine, tyrosine and tryptophan biosynthesis	4	2	1.0638	0.021006	0.65117	0.14844
Arginine and proline metabolism	36	2	1.0638	0.026998	0.78295	0.14844
Histidine metabolism	16	3	1.0638	0.053452	1.0	0.14844
beta-Alanine metabolism	21	2	1.0638	0.062471	1.0	0.14844
Pyruvate metabolism	23	3	1.0638	0.065224	1.0	0.14844
Glycolysis / Gluconeogenesis	26	2	1.0638	0.065464	1.0	0.14844
Glyoxylate and dicarboxylate metabolism	31	2	1.0638	0.065464	1.0	0.14844
Galactose metabolism	27	1	1.0638	0.066131	1.0	0.14844
Starch and sucrose metabolism	18	1	1.0638	0.066131	1.0	0.14844
Neomycin, kanamycin and gentamicin biosynthesis	2	1	1.0638	0.066131	1.0	0.14844
Primary bile acid biosynthesis	46	1	1.0638	0.067472	1.0	0.14844
Taurine and hypotaurine metabolism	8	1	1.0638	0.067472	1.0	0.14844
Propanoate metabolism	21	1	1.0638	0.089638	1.0	0.18488
Citrate cycle (TCA cycle)	20	3	1.0638	0.10592	1.0	0.19492
Glycerophospholipid metabolism	36	2	1.0638	0.10632	1.0	0.19492
Valine, leucine and isoleucine biosynthesis	8	1	1.0638	0.19959	1.0	0.34639
Ubiquinone and other terpenoid-quinone biosynthesis	18	1	1.0638	0.20993	1.0	0.34639
Valine, leucine and isoleucine degradation	39	2	1.0638	0.25565	1.0	0.40173
Alanine, aspartate and glutamate metabolism	28	5	1.0638	0.47617	1.0	0.69508
Cysteine and methionine metabolism	33	2	1.0638	0.50786	1.0	0.69508
Lysine degradation	30	1	1.0638	0.54412	1.0	0.69508
Biotin metabolism	10	1	1.0638	0.54412	1.0	0.69508

[1]Total: total metabolites found in metabolic pathways;

[2] Hits: total study metabolites found in metabolic pathways;

[3] Expected: Number of expected hits

[4] The *p* value is used to evaluate the statistical significance of the association between metabolites or metabolic pathways. The lower the *p* value, the greater the evidence of a significant association

[5]The Holm correction was used to adjust p-values in multiple analyses, reducing the risk of false positives.

[6] FDR: false discovery rate.

Supplementary Figure S1

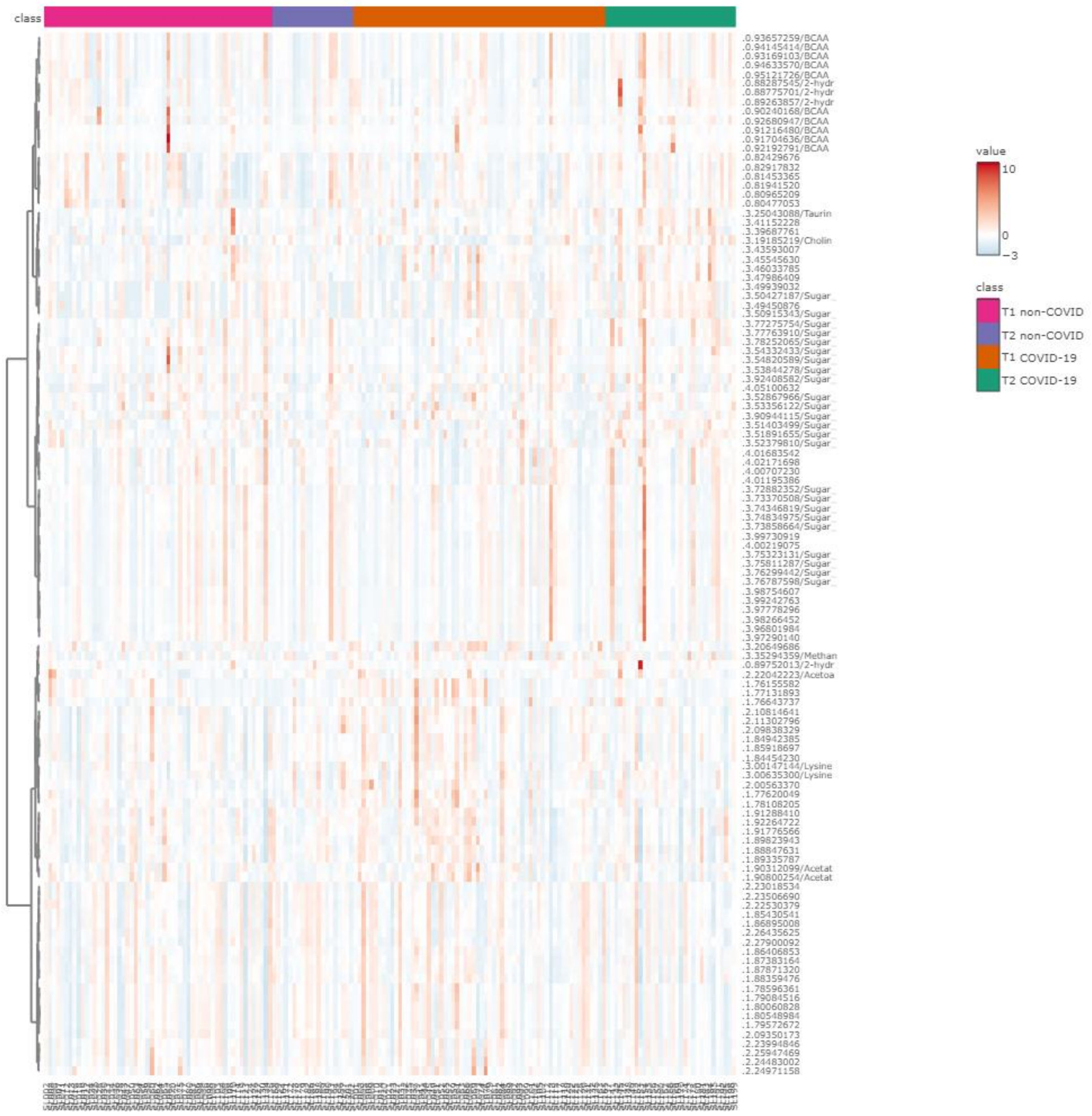

Figure S1. The heatmap of the relative changes in metabolite abundance in T1 and T2 of individual samples in non-COVID and COVID-19 groups. Red hues indicate upregulated metabolites, while blue hues indicate downregulated metabolites in the salivary samples of the study subjects. The classes were defined in the following colors: pink: T1 non-COVID, purple: T2 non-COVID, red: T1 COVID-19 and green: T2 COVID.

Supplementary Figure S2

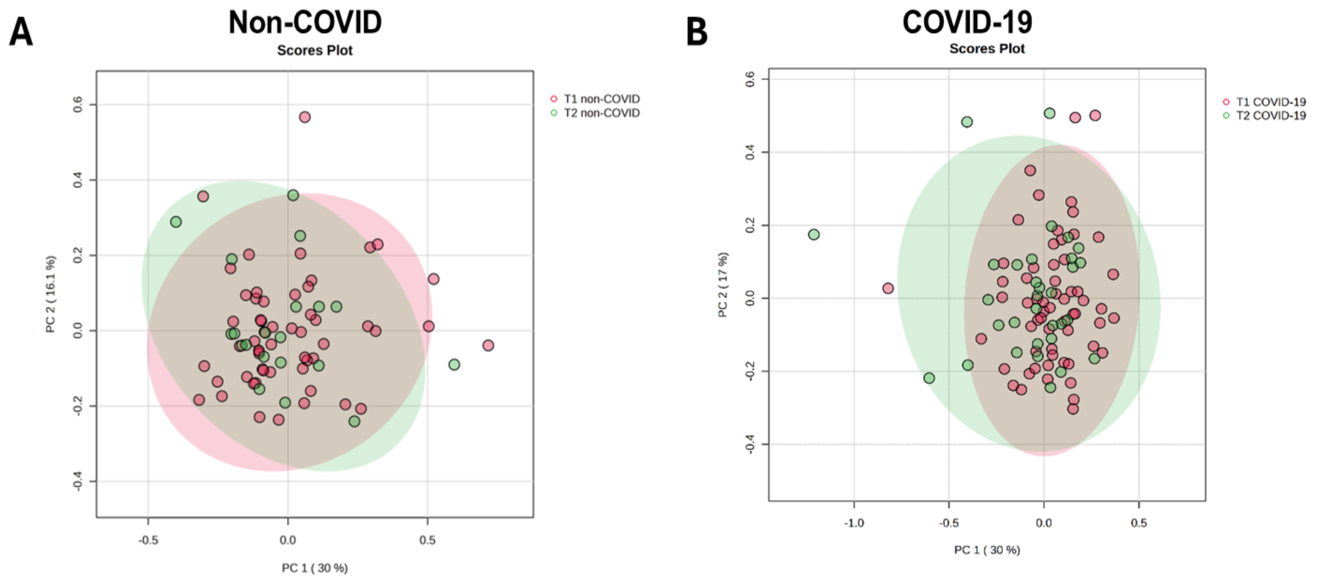

Supplementary Figure S2. The PCA and loading factors plot highlights the metabolic profile between T1 and T2 of non-COVID and COVID-19 groups according to ^{1}H NMR-based metabolomics. PCA plot shows similar profile in the (A) T1 x T2 non-COVID and (B) T1 x T2 COVID-19 group.

Supplementary Figure S3

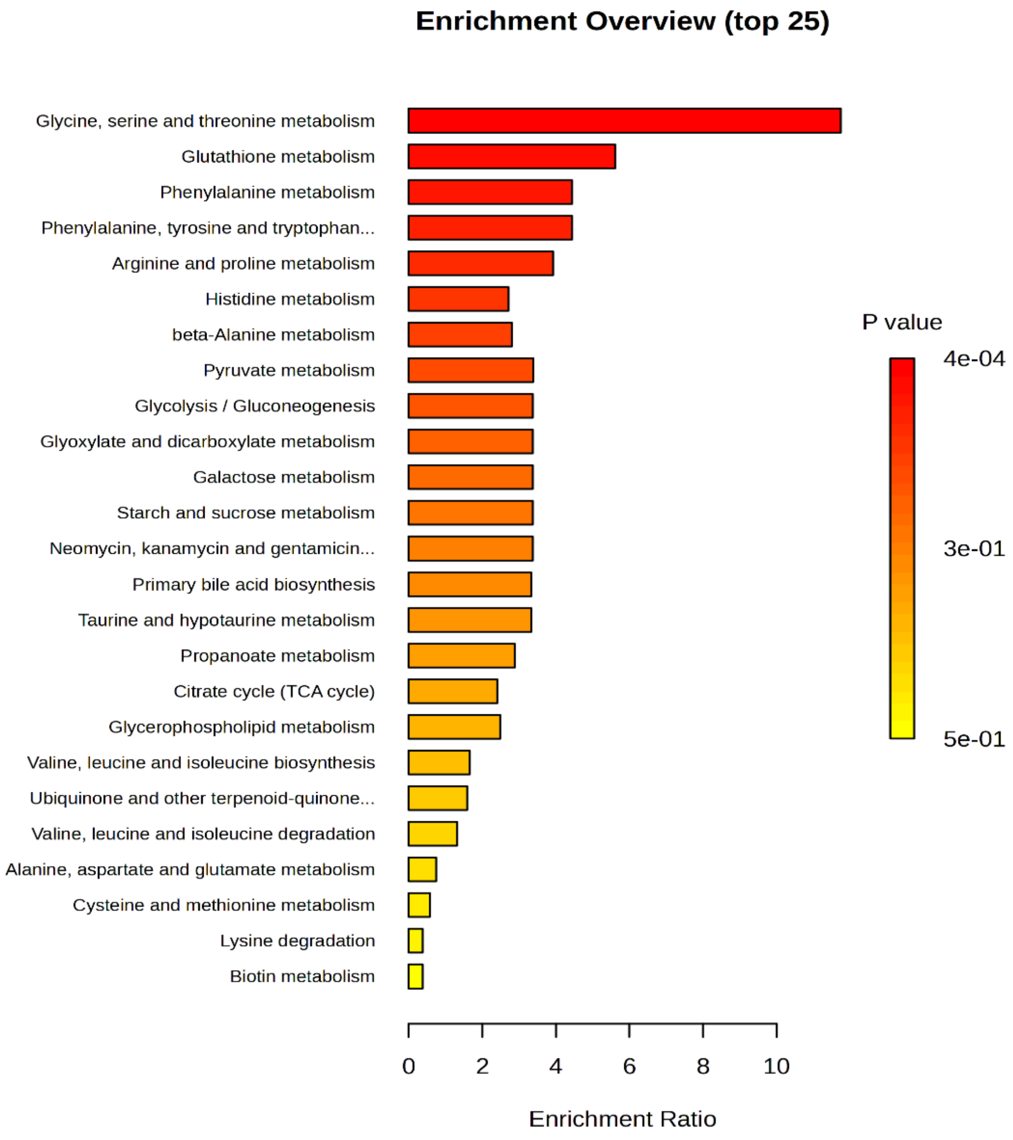

Figure S3. Metaboanalyst enrichment shows 25 pathways associated with the acute and post-acute phase of the COVID-19 group. The enrichment rate represents the proportion of observed metabolites within a specific metabolic pathway relative to the expected number of metabolites.

Supplementary Figure S4

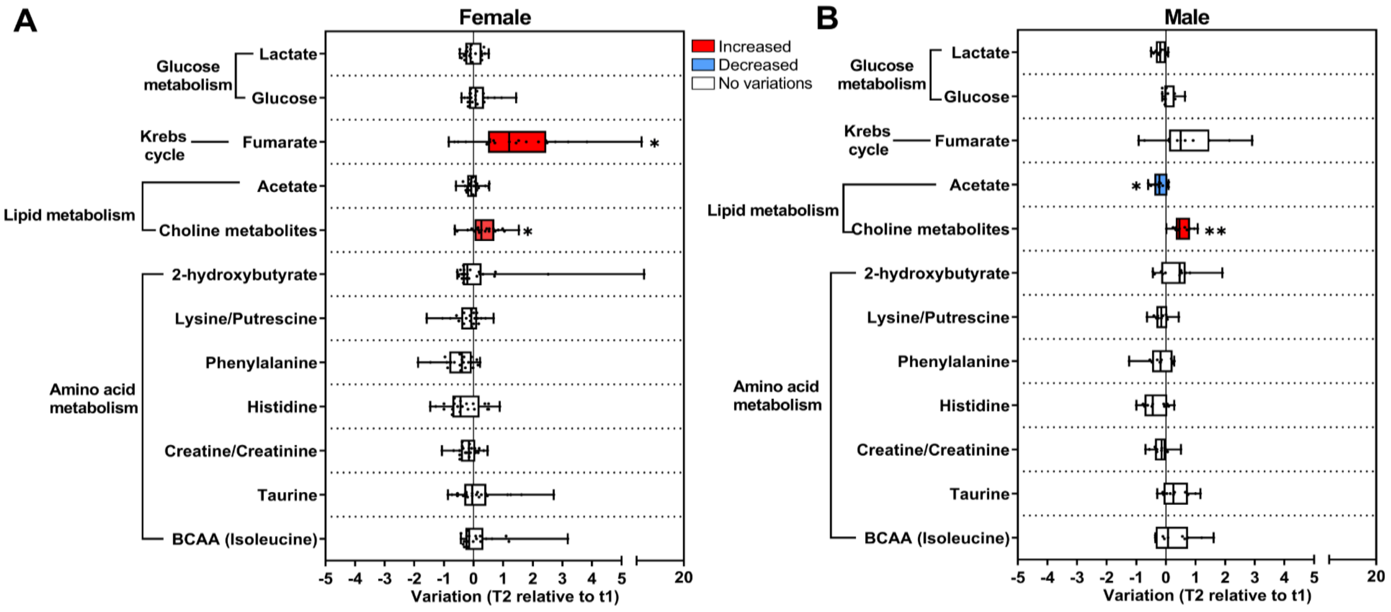

Supplementary Figure S4. Metabolic profile in the COVID-19 group was not discriminated by sex. The range (mean T2/T1) was calculated based on the metabolite intensities within each group. Longitudinal changes in metabolites are presented as the ratio T2 to T1 calculated from metabolites intensity according to ^{1}H NMR metabolomics. Red bars: higher metabolite content in T2; blue bars: lower metabolite content in T2; white bars: no differences between T1 and T2 phases. (A) female (T1 n=35; T2 n=21); (A) male (T1 n=28; T2 n=11). *P<0.05, **P<0.01***P<0.001, according to Kruskal-Wallis test.
